# Supplementary material for: Modeling glioblastoma heterogeneity as a dynamic network of cell states
Source: Mol Syst Biol. 2021 Sep 16;17(9):e10105. doi: 10.15252/msb.202010105 (PMC8444284; doi:10.15252/msb.202010105)
Supplement: Supplementary file 6 — Source Data for Figure 5 [file MSB-17-e10105-s004.zip › Figure5A_sourcedata/GSEA_3017/hallmarks_stateF.GseaPreranked.1621934628066/HALLMARK_CHOLESTEROL_HOMEOSTASIS.html]

Details for gene set HALLMARK\_CHOLESTEROL\_HOMEOSTASIS[GSEA]

|  || Dataset | state33017 |
| Phenotype | NoPhenotypeAvailable |
| Upregulated in class | na\_pos |
| GeneSet | HALLMARK\_CHOLESTEROL\_HOMEOSTASIS |
| Enrichment Score (ES) | 0.40961677 |
| Normalized Enrichment Score (NES) | 1.6316004 |
| Nominal p-value | 0.034798536 |
| FDR q-value | 0.047741737 |
| FWER p-Value | 0.465 |
Table: GSEA Results Summary

  

Fig 1: Enrichment plot: HALLMARK\_CHOLESTEROL\_HOMEOSTASIS      
 Profile of the Running ES Score & Positions of GeneSet Members on the Rank Ordered List

  

| PROBE | GENE SYMBOL | GENE\_TITLE | RANK IN GENE LIST | RANK METRIC SCORE | RUNNING ES | CORE ENRICHMENT || 1 | CLU |  |  | 5 | 1.852 | 0.1566 | Yes |
| 2 | LGALS3 |  |  | 16 | 1.261 | 0.2603 | Yes |
| 3 | PNRC1 |  |  | 23 | 1.070 | 0.3494 | Yes |
| 4 | LPL |  |  | 59 | 0.874 | 0.4087 | Yes |
| 5 | TM7SF2 |  |  | 225 | 0.570 | 0.3837 | Yes |
| 6 | HMGCS1 |  |  | 271 | 0.537 | 0.4096 | Yes |
| 7 | ATXN2 |  |  | 477 | 0.442 | 0.3557 | No |
| 8 | CPEB2 |  |  | 588 | 0.401 | 0.3409 | No |
| 9 | S100A11 |  |  | 692 | 0.373 | 0.3268 | No |
| 10 | SC5D |  |  | 737 | 0.362 | 0.3381 | No |
| 11 | HSD17B7 |  |  | 791 | 0.349 | 0.3443 | No |
| 12 | CD9 |  |  | 1070 | 0.297 | 0.2453 | No |
| 13 | LGMN |  |  | 1110 | 0.291 | 0.2528 | No |
| 14 | CTNNB1 |  |  | 1190 | 0.280 | 0.2414 | No |
| 15 | STARD4 |  |  | 1254 | 0.274 | 0.2367 | No |
| 16 | GLDC |  |  | 1425 | 0.255 | 0.1824 | No |
| 17 | ACAT2 |  |  | 1512 | -0.263 | 0.1665 | No |
| 18 | TMEM97 |  |  | 1629 | -0.293 | 0.1397 | No |
| 19 | SQLE |  |  | 1863 | -0.396 | 0.0693 | No |
| 20 | FABP5 |  |  | 2033 | -0.523 | 0.0385 | No |
| 21 | TNFRSF12A |  |  | 2116 | -0.694 | 0.0614 | No |
Table: GSEA details [plain text format]

  

Fig 2: HALLMARK\_CHOLESTEROL\_HOMEOSTASIS: Random ES distribution      
 Gene set null distribution of ES for **HALLMARK\_CHOLESTEROL\_HOMEOSTASIS**
